# A Double-Layer Hydrogel Dressing with High Mechanical Strength and Water Resistance Used for Drug Delivery

**DOI:** 10.3390/molecules28020499

**Published:** 2023-01-04

**Authors:** Fangzhe Liu, Zihan Wang, Hui Guo, Haichao Li, Yulan Chen, Shuang Guan

**Affiliations:** 1 School of Chemistry and Life Science, Changchun University of Technology, Changchun 130012, China; 2 Advanced Institute of Materials Science, Changchun University of Technology, Changchun 130012, China; 3 State Key Laboratory of Supramolecular Structure and Materials, College of Chemistry, Jilin University, Changchun 130012, China

**Keywords:** β-cyclodextrin, hydrogel dressing, double-layer hydrogel, drug release model

## Abstract

Hydrogel dressings provide a moist wound healing environment, absorb the exudates of the wound, and have better biocompatibility than traditional dressings. However, it is still difficult to meet the needs of modern medicine due to the defects in drug burst release, weak mechanical strength, and poor water retention. To solve these problems, we developed a double-layer (DL) hydrogel based on β-cyclodextrin polymer (β-CDP), poly(vinyl alcohol) (PVA), and carboxymethyl cellulose sodium (CMC) via a layer-by-layer method. Inspired by natural coconut, this hydrogel consisted of a drug release layer (DRL) and a mechanical support layer (MSL). In our design, the introduction of β-CDP into the DRL slowed the drug release rate of the DL hydrogel. Furthermore, the mechanical strength of the hydrogel was improved by immersing the MSL in a calcium chloride/boric acid solution. Combining these two layers, the tensile strength and elongation at break of the DL hydrogel reached 1504 kPa and 400%, respectively. More interestingly, the release mechanism of DL hydrogel conformed to the diffusion–relaxation–erosion model, which was different from traditional hydrogel dressings. Therefore, the as-prepared DL structure represents a feasible solution for fabricating high-performance mechanical hydrogel dressings with sustained drug release properties, and the DL hydrogel has potential to be used for medical dressings applied in daily life.

## 1. Introduction

Medical dressings are essential medical supplies in daily life. They can create a healing environment, protect the wound’s surface from bacterial infection, and repair skin damage during wound care. The drug is placed into the dressing to deliver medication to the wound site consistently and continuously over an extended period of time without the need for frequent dressing changes. There are two types of medical dressing: traditional medical dressing and advanced medical dressing. Traditional medical dressings include cotton batting, gauze, and bandages. Traditional dressings can protect wounds but do not meet modern medical dressings’ standards regarding water permeability, bacterial infection prevention, and biocompatibility. Compared with the traditional medical dressing, the new medical dressing can better maintain the moisture of the wound location and provide a suitable environment for wound recovery. With the development of society, biomedical hydrogel dressings with a 3D network structure and high water absorption ability emerged at a historic moment, and the research of hydrogel dressings has become a research hotspot. Because of its adaptable physical and chemical properties and structural similarity with the extracellular matrix, it has a wide range of application prospects [1,2,3,4]. In earlier studies, researchers directly added drugs into hydrogels to achieve drug release. However, the burst release of drugs could produce many adverse effects. Therefore, many drugs were loaded into functionalized carriers to reduce the drug release rate and achieve controlled drug release [5]. For example, Vivian F. Lotfy et al. fabricated a chitosan derivative hydrogel for drug loading and investigated the drug release mechanism [6]. Seonmok Kim et al. designed a polysaccharide hydrogel with outstanding biocompatibility and self-healing properties. The hydrogel showed apH-responsive drug release behavior [7]. However, there are many original weaknesses of single-layer hydrogels, such as weak mechanical strength and low water retention, which restrict their practical application [8]. Hence, more complex hydrogel structures are required to solve these problems [9].

Nature is a great treasure for human beings, and many of our designs and products are inspired by it. We can effectively promote our understanding of nature and solve real-life problems through imitation and learning from nature. With a hard shell and pulp content, coconut can protect the inner coconut juice [10]. There is no doubt that scientists have applied this exciting structure in their research. Nina Graupner et al. fabricated a simple, layered, cellulose fiber-reinforced polylactide (PLA) made by stacking layers reinforced with fibers of different mechanical properties [11]. The bionic, asymmetrically graded composites showed excellent flexural strength and modulus compared to the reference samples. Inspired by the layered structure of coconut, multifunctional double-layer (DL) hydrogels are promising in the field for their excellent mechanical strength and sustained drug release ability. However, the research on DL hydrogels has mainly focused on the deformation caused by the transformation of isotropy to anisotropy. For instance, based on spiropyran molecular design, Li et al. proposed a photoactive DL hydrogel that could rapidly respond to visible light and lead to complex but predictable bio-inspired shape changes [12]. He et al. presented bilayer poly(N-isopropylacrylamide)/graphene oxide (PNIPAM/GO) hydrogels with dual thermo- and near-infrared (NIR)-responsive deformation [13]. Only a few researchers have applied DL hydrogel in medical dressings. Javad Tavakoli et al. prepared a PVA-PAA DL hydrogel dressing possessing ultimate tensile strength, modulus, and elongation at break through a layer-by-layer process [14]. To the best of our knowledge, current research about the drug release kinetics of hydrogels is mostly focused on drug release from monolayer hydrogels and hydrogel spheres, but the release behavior of DL hydrogel dressings still needs further investigation [15,16,17].

Therefore, we have designed a favorable DL hydrogel dressing containing a drug release layer (DRL) and a mechanical support layer (MSL). The DL hydrogel can provide outstanding mechanical strength and sustained drug release ability by combining the two layers with different functions. The DRL could achieve the drug’s slow release by adding a β-cyclodextrin polymer (β-CDP), and the MSL could improve the gel’s mechanical strength by immersing it in a salt solution. In this work, β-cyclodextrin (β-CD) was selected as a drug carrier precursor due to its biological safety and ability to encapsulate guest molecules by the interaction of host and guest [18]. To reduce the limitation caused by poor water solubility, we prepared β-CDP through β-CD monomer and epichlorohydrin (EPI), and selected it as a drug carrier because of its solubility and the drug loading properties [19]. Diclofenac sodium (DS) was introduced into the DRL as a model drug to discuss the unique drug release model and explain its mechanism. Through characterization, the DL hydrogel’s mechanical strength, water retention rate, and drug release time could reach 1504 kPa, 67.31% (48 h), and 8 h, respectively. Therefore, the bionic DL hydrogel could serve as a hydrogel dressing for practical application, which is beneficial for developing hydrogels in medical materials.

## 2. Results and Discussion

### 2.1. Synthesis and Characterization of β-CDP

**Scheme 1:**
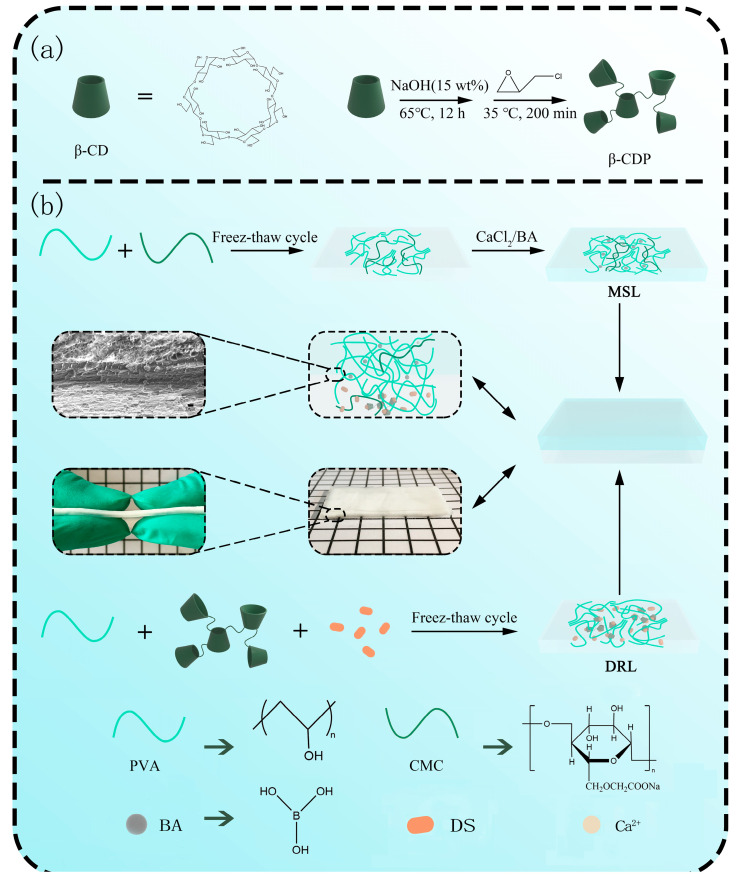
Schematic illustration of (**a**) preparation of β-CD polymers (β-CDP) and (**b**) preparation of the DL hydrogel.

As shown in Scheme 1a, β-CDP was prepared by the reaction between the hydroxyl hydrogen of β-CD and the epoxy group of EPI in the alkaline environment. Under the catalysis of NaOH, the desired polymer was obtained by epoxy group opening and closing reactions.

**Figure 1:**
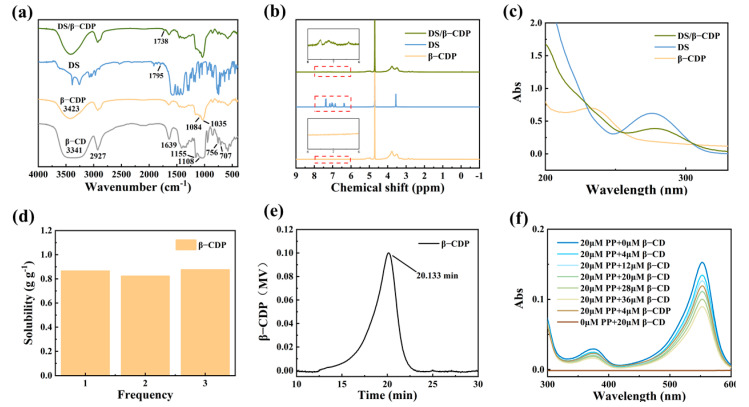
Structure and properties of β-CDP. (**a**) FT-IR spectra of β-CD, β-CDP, DS, and DS/β-CDP. (**b**) ^1^H NMR spectra of β-CDP, DS, and DS/β-CDP. (**c**) UV–Vis spectra of β-CDP, DS, and DS/β-CDP. (**d**) Solubility and (**e**) GPC analysis of β-CDP. (**f**) UV–Vis spectra of PP/β-CDP and PP/β-CD with different content.

The FT-IR spectra of β-CD, β-CDP, DS, and DS/β-CDP are shown in Figure 1a. In the spectrum of β-CDP, the peak corresponding to the -OH had a significantly blue shift from 3341 cm^−1^ to 3423 cm^−1^ and the new peaks of C-O-C (1035 cm^−1^ and 1084 cm^−1^) appeared, indicating that the β-CDP had been successfully prepared through the ring opening and closing reactions. In the spectra of DS/β-CDP, an extra sharp band at 1738 cm^−1^ could be assigned to the stretching vibration of C=O in the carboxyl group of DS, confirming that DS was successfully loaded into β-CDP.

Figure 1b shows the ^1^H NMR spectra of β-CDP, DS, and DS/β-CDP. Compared with β-CDP, the spectrum of DS/β-CDP showed new characteristic peaks at the range of 6.0–8.0 ppm derived from the protons of DS, indicating that DS had been successfully loaded in β-CDP. In addition, the UV–Vis absorption spectra of these samples are exhibited in Figure 1c. After the loading of DS, a new absorption peak at 275 nm could be observed in the spectrum of DS/β-CDP, further indicating that the DS-loaded inclusion complex was prepared.

Since the solubility and activity (the active monomer quantity in β-CDP) of β-CD and β-CDP can affect the drug loading rate, the solubility and activity changes of β-CD before and after polymerization were detected and compared. As shown in Figure 1d, the solubility of the β-CDP significantly increased to 0.855 g g^−1^ in an aqueous solution at room temperature (25 °C), which was much higher than the solubility of β-CD (0.0184 g g^−1^) [20]. Then, the degree of polymerization was determined by the GPC by detecting the molecular weight distribution of β-CDP. The results showed that the retention time of β-CDP (Tr) was 20.13 min (Figure 1e), and the average molecular weight and the degree of polymerization of β-CDP were calculated to be 5864 g mol^−1^ and 5, respectively. According to the polymerization, the activity of β-CDP could still be maintained at 76.77% through the measurement of the PP probe technology (Figure 1f). Finally, through calculations (Equation (3)), the drug loading capacity of β-CDP was higher than that of β-CD, which indicated that the influence of solubility was greater than the activity on the drug loading rate, so the β-CDP could effectively improve the solubility of the drug in water.

### 2.2. Preparation and Mechanical Property Analysis of MSL

**Figure 2:**
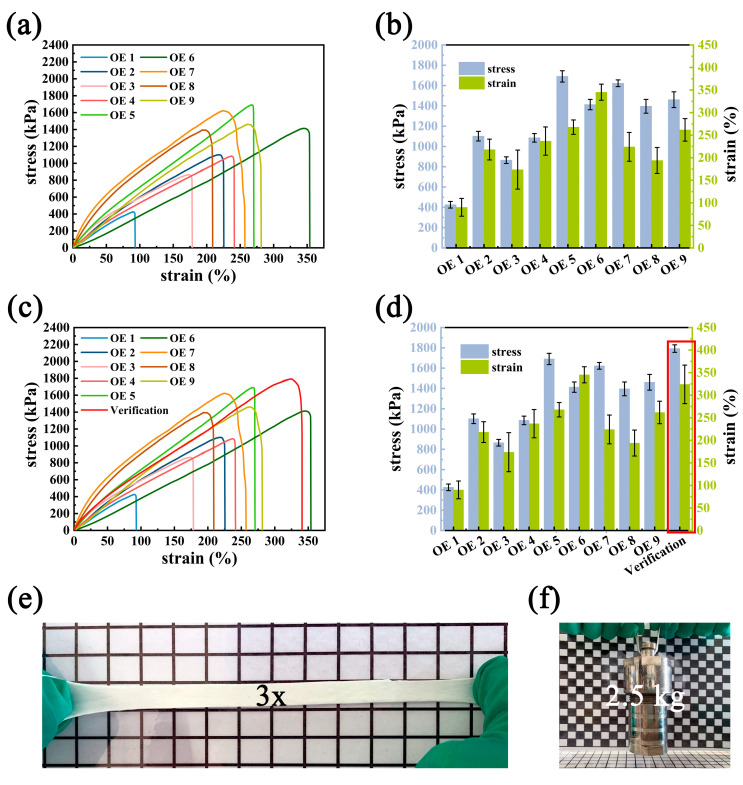
Mechanical performance. (**a**) Tensile stress–strain curves, and (**b**) the corresponding stress and strain of MSL hydrogel with different component ratios via orthogonal experimental design. (**c**) Verification of the optimum MSL and (**d**) the corresponding stress–strain curves. Optical images showing (**e**) the excellent stretching of MSL hydrogel and (**f**) the loading demonstration of MSL hydrogels.

Based on the preliminary study, an L9 (3^3^) orthogonal experiment including three principal factors (the dosage of PVA and CMC, soaking time) was designed to obtain the desired MSL. The stress strength was detected as an indicator to evaluate the mechanical property of MSL. The results of the orthogonal experiment (Figure 2a,b) and the variance analysis (Table S1) indicated that the influence degree of the main factors in mechanical strength of hydrogels was as follows: dosage of PVA > dosage of CMC > soaking time. MSL could exhibit the best mechanical strength when the experimental conditions entailed adding 3.5 g of PVA and 0.2 g of CMC and soaking 1.5 h (A_3_B_2_C_3_). As shown in Figure 2c,d, the mechanical strength of hydrogel with the optimal ratio was strictly higher than other groups (Figure 2c,d). The optimal MSL with a length of 4 cm could be easily stretched to 12 cm and lift a 2.5 kg steel reaction kettle without any breakage (Figure 2e,f). These results suggest that the prepared MSL could endow the DL hydrogel with excellent mechanical properties to expand its practical application.

### 2.3. Preparation and Drug Release Behavior Analysis of DRL

**Figure 3:**
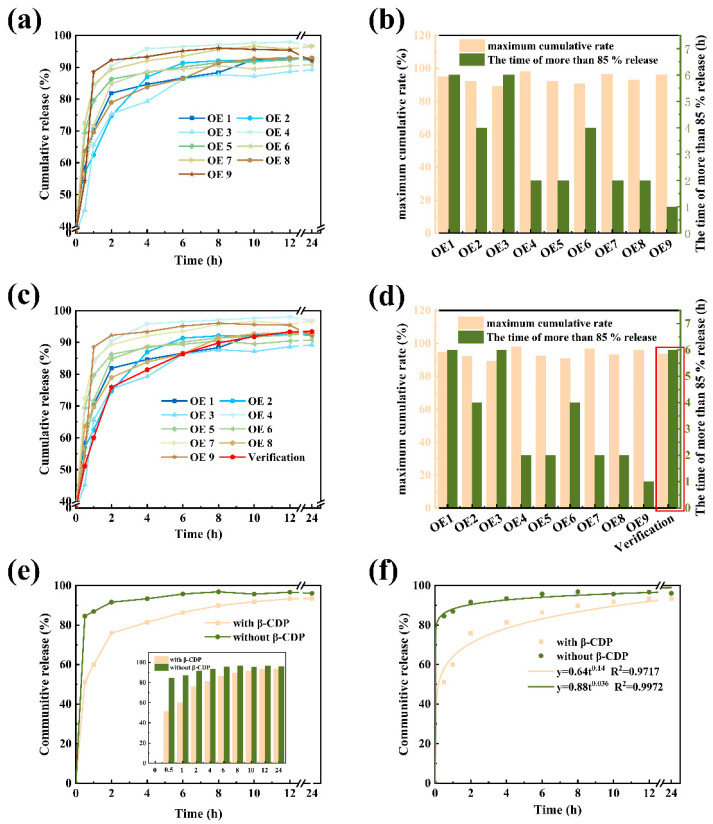
Drug release behavior and analysis of DRL. (**a**) Release behavior of DRL via orthogonal experimental design in pH = 7.4 PBS at 37 °C and (**b**) the corresponding release behavior with different component. (**c**) Verification of the optimum release behavior and (**d**) the corresponding release behavior of DRL. (**e**) Verification of the release behavior of DRL with or without β-CDP and (**f**) the drug release kinetics fitting analysis.

Similarly, another L9 (3^4^) orthogonal experiment including four principal factors (the dosage of PVA, β-CDP and DS, freeze–thaw cycle times) was conducted to obtain the desired DRL. The maximum cumulative release ratio of the model drug and the time of more than 85% release were considered as two test indexes to evaluate the sustained release ability of DRL. From the results (Figure 3a,b, Equation (1), and Table S2), the order of the main influencing factors for drug release was as follows: the dosage of DS > PVA > β-CDP > the freeze–thaw cycle times. When adding 1.5 g PVA, 1.0 g β-CDP, 0.2 g DS, and going through three freeze–thaw cycles (A_1_B_1_C_1_D_3_), the sample DRL exhibited the best mechanical strength (Figure 3c,d). Then, the sustained release behavior of DRL with or without β-CDP was investigated (Figure 3e). The DRL with β-CDP could prolong the drug release time, demonstrating that adding β-CDP could endow the hydrogel with the capacity to release the drug slowly.

(1) $$score=\frac{releasing\;amount}{2}+\frac{\frac{time}{24}}{2}$$

Finally, we applied the release kinetics data of DRL to several typical drug release models (Equations (5)–(7)). We found the release mechanism of the DRL in PBS buffer solution (pH = 7.4) consistent with the Ritger–Peppas model (Figure 3f). The rate constant “k” and diffusion index “n” were obtained from the above formula (Table 1), and the diffusion index n was 0.14 (<0.45). The above results revealed that the drug releasing mechanism belonged to Fick diffusion [21]. Because the DRL was prepared by physical crosslinking, the gel molecular chain relaxed quickly and failed to limit the release of the drug [22,23]. Hence, the drug diffused freely from the gel to the external environment by the concentration gradient. Apart from this, the drug in β-CDP would also be released slowly, prolonging the release time.

### 2.4. Preparation and Characterization of DL Hydrogel

We combined MSL and DRL to form a DL hydrogel dressing and then comprehensively investigated its mechanical properties, water retention capacity, skin adhesion property, and drug sustained release property.

**Figure 4:**
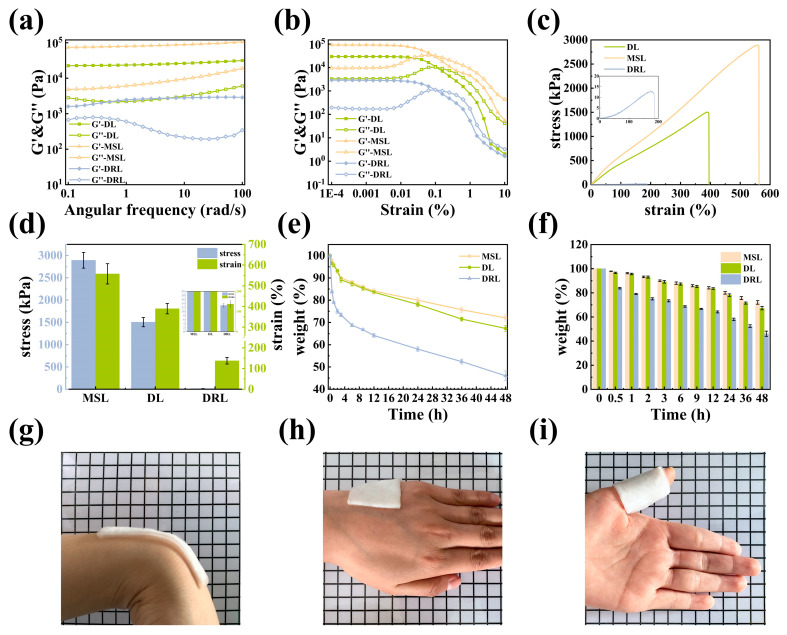
Mechanical performance of the DL hydrogel. (**a**) The oscillation angular frequency sweep rheological measurement. (**b**) The oscillation strain amplitude sweep rheological measurement. (**c**) Tensile stress–strain curves and (**d**) the corresponding stress and strain of MSL, DL, and DRL hydrogels. (**e**) Water retention curves and (**f**) the corresponding weight of MSL, DL, and DRL hydrogels in a chamber at room temperature. Adhesive to (**g**) wrist and (**h**) hand. (**i**) Wrapped around the finger.

The mechanical properties of the DL hydrogels were evaluated by detecting their rheological and tensile stress–strain curves. Storage modulus (G′, filled symbols) and loss modulus (G″, empty symbols) of hydrogels were measured as a function of angular frequency (0.1–100 rad/s) and strain sweep (0.0001–10%) (Figure 4a,b). Over the entire frequency range, all G′ values of hydrogels were higher than the G″ values, meaning that all hydrogels were in the elastic solid gel state instead of the fluidic sol state [24]. Obviously, the G′ of MSL was much higher than the G′ of DL and DRL, which meant the MSL could effectively provide enough mechanical strength for the DL hydrogel. Meanwhile, in the linear viscoelastic region of strain from 0.0001% to 0.01%, the G′ of the DL hydrogel was always much higher than others, owing to the formation of boron ester bonds in hydrogels. When the strain was more than 0.01%, values of G′ decreased dramatically, and the curves of each hydrogel had a crossover at a strain near 0.1%, which suggested that the gel network was totally broken and converted into a sol state at this strain [25]. Next, the tensile stress–strain curves of the MSL, DL, and DRL hydrogels were investigated (Figure 4c,d). The mechanical strength of MSL and DL hydrogel could reach 2892 kPa and 1504 kPa, respectively, while the mechanical strength of the DRL was only 12.81 kPa. Therefore, all these results demonstrated that the mechanical strength of DL hydrogel was strengthened by combining MSL with DRL [26].

Materials with suitable water retention properties are essential in wound dressings because they provide a moist microenvironment to facilitate wound healing. Therefore, we conducted a series of contrast experiments to evaluate this property of our hydrogel (Equation (8), Figure 4e,f). The results indicated that these hydrogels exhibited similar characteristics: on the one hand, for DRL, DL, and MSL, as the standing time reached 48 h, their water retention rate decreased from 100% to 46.05%, 67.31%, and 72.06%, respectively; on the other hand, when the water retention rate dropped to 70%, the DRL, DL, and MSL needed 5 h, 40 h, and more than 48 h, respectively. From the above two points, we can conclude that the introduction of MSL could effectively improve the water retention property of the DL hydrogel due to the existence of denser cross-linked networks for the reduction in water loss in the MSL.

As a dressing, excellent skin adhesion was also essential for hydrogels. As shown in Figure 4g–i, the DL hydrogel could attach to the wrist, hand, and finger without falling, demonstrating that it had suitable adhesion ability due to the electrostatic and hydrogen bond interactions between the hydrogel and skin [27].

**Figure 5:**
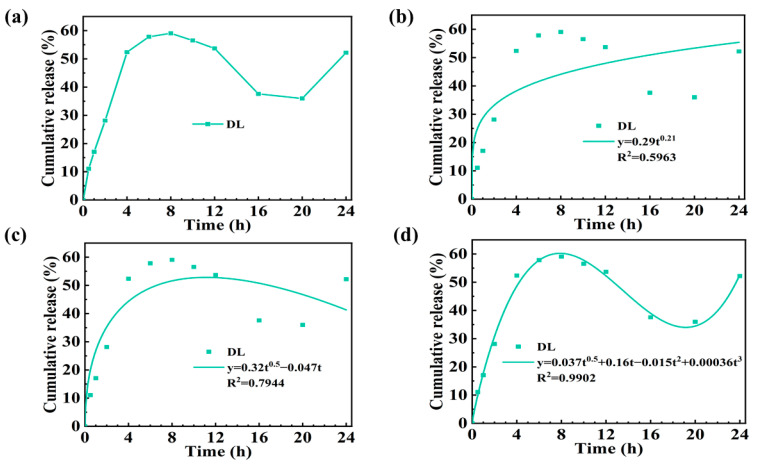
The cumulative release curve of DS from DL hydrogel at pH 7.4. (**a**) Drug release curve for DL hydrogel. Drug release kinetics model analysis: (**b**) Korsmeyer–Peppas model, (**c**) Peppas–Sahlin model (diffusion–relaxation model), and (**d**) diffusion–relaxation–erosion model.

Then, we detected the DS sustained release ability of DL hydrogel. Figure 5a displays the drug release profile of our DS-loaded DL hydrogels. The DL hydrogel’s sustained release time could reach 8 h, and its total drug releasing amount was lower than DRL (59.1% < 93.4%). Unlike DRL, the drug release amount decreased sharply after 8 h and rose again at 20 h, forming an “S”-shaped curve and attracting our attention. As far as we know, the drug release rate of most typical hydrogels was constant when the cumulative release reached the maximum [28,29,30,31]. Therefore, we speculated that the drug release mechanism of the DL hydrogel was different from traditional hydrogel dressings.

To explore the drug release mechanism of DL hydrogel, we applied the release kinetics data of the DL hydrogel to several typical drug release models (Figure 5b–d), and the fitting parameters are shown in Table 2. Various mathematical models describing drug release provided insights into the mechanism of drug delivery. While diffusion was a prevailing mechanism for drug release from polymer networks, swelling and erosion might take over in some polymeric carriers. According to the results (Table 2), Ritger–Peppas and Peppas–Sahlin mathematical models indicated diffusion and diffusion–relaxation controlled systems, which failed to describe DS release from the DL hydrogel. Based on the drug release mechanism of DRL, the m in the diffusion–relaxation–erosion model was determined as 0.5 from Table 3 [32] ^1 2^. Compared to other models, this semi-empirical equation’s corresponding regression coefficient (R^2^ = 0.9902) was the closest to 1, so we concluded that the drug release behavior of the DL hydrogel obeyed the diffusion–relaxation–erosion model. The reasons for this phenomenon can be summarized as follows: compared with the drug release of monolayer hydrogel, the DL hydrogel not only released drugs directly to the external solution but also diffused part of drugs from the DRL to the MSL, and then released them to the solution. Therefore, the DL hydrogel’s release time would be prolonged, but the release rate would decrease. At the same time, because of the swelling phenomenon caused by the double-layer structure of DL hydrogel, it could lead to a specific drug absorption. Then, with the prolongation of the soaking time, the DRL of the hydrogel would be corroded to release more drugs and the MSL would be swollen, so that some of the reabsorbed drugs would release again [22].

Finally, we compared our DL hydrogel dressing with other dressings (such as layered dressing and hydrogel microspheres, etc.) reported in the field of drug delivery in recent years (Table 4). It could be concluded that traditional hydrogels had poor performance in mechanical strength and water retention, especially in the field of hydrogel dressing. Unlike previous works, we first applied the DL structure to manufacturing hydrogel dressing to simplify the design of a multifunctional single-layer dressing, endowing the hydrogel with better mechanical strength (G′ = 2.5 × 104 Pa) as well as water retention (48 h, 67%). This structure could prolong the drug release process simultaneously. Benefiting from the DL structure, this drug release model of hydrogel was different from previous work (diffusion–relaxation–erosion model rather than the Ritger–Peppa model). In summary, our design could equip hydrogel dressings with many excellent properties and broaden the practical application range.

## 3. Materials and Methods

### 3.1. Materials

Poly (vinyl alcohol) 1799 (PVA, Shanghai, Aladdin, 98–99%), β-cyclodextrin (β-CD, Aladdin, 98%), (±)-Epichlorohydrin (EPI, Aladdin, AR), acetone (ACE, Beijing Chemical Works, AR), carboxymethyl cellulose sodium (CMCS, Aladdin, USP), diclofenac sodium (DS, Aladdin, ≥99%), calcium chloride anhydrous (CaCl_2_, Tianjin Beilian Co. Ltd., ≥96%), boric acid (BA, Aladdin, GR), and phenolphthalein (PP, Aladdin, 98%) were used. Deionized water was used in all the experiments.

### 3.2. Characterization

The Fourier transform infrared (FT-IR) spectra of β-CD, β-CDP, DS, and DS/β-CDP were collected in the range of 400–4000 cm^−1^ using a Fourier transform infrared spectrometer (Nicolet-IS50, Thermofisher Nicolet, New York, StateofNewYork, USA) at room temperature. The ^1^H NMR spectra of DS, β-CDP, and DS/β-CDP were recorded on ^1^H NMR (9.4 T Bruker Avance III 400 MHz NMR spectrometer, Billerica, Massachusetts, Germany)at 25 °C in D_2_O. The UV–Vis spectra of DS, β-CDP, and DS/β-CDP were detected using a UV–Vis spectrophotometer (Cary 5000, Agilent, Palo Alto, CA, USA).

### 3.3. Synthesis of β-CD Polymers (β-CDP) and DS/β-CDP

The preparation was carried out according to the previously reported procedure [38]. Briefly, 2.25 g of NaOH was dissolved in 15 mL of deionized water (0.15% w/w) followed by adding 10 g of β-CD, and the mixture was stirred at 65 °C all night to activate the hydroxyl of β-CD. Then, 6 mL of crosslinker (EPI) was added dropwise into the β-CD solution to crosslink β-CD. After 200 min, the reaction was terminated by adding acetone, and then the underlying fluid was collected and diluted with deionized water. The pH was adjusted to neutral by using hydrochloric acid. Finally, the product was purified by dialysis (molecular weight cut off 8–14 kDa) against deionized water for 7 days. The final product, β-CDP, was obtained through freeze-drying of the dialysate. The preparation process was schematically shown in Scheme 1a.

For drug loading, 80 mg DS was dissolved in 5 mL of deionized water to form a drug solution. Then, 295 mg β-CDP was added to the prepared solution and stirred overnight. Finally, the above solution was dialyzed (molecular weight cut off 1 kDa) against deionized water for 24 h and then lyophilized to obtain the drug-loaded DS/β-CDP.

### 3.4. Preparation of Double-Layer (DL) Hydrogel

Mechanical support layer (MSL): The L9 (33) orthogonal experiments were designed to optimize the MSL preparation conditions. PVA (2.5–3.5 g) and CMC (0.1–0.3 g) powders were co-dissolved in 20 mL deionized water at 95 °C and stirred for 1 h. After cooling to room temperature (25 °C), the solution was injected into a reaction mold (100 × 100 × 2 mm^3^). Then, the reaction mold was put into the refrigerator and frozen at −20 °C for 3 h. After that, the mold was removed and balanced for 1 h at room temperature to obtain the PVA/CMC hydrogels. Finally, 4 g calcium chloride and 4 g boric acid were co-dissolved in 100 mL deionized water to obtain the soaking solution under magnetic stirring. The PVA/CMC hydrogels were immersed in this solution for 0.5–1.5 h to obtain the MSL. The amounts of each polymer monomer and soaking time are shown in Table 5.

Drug release layer (DRL): The L9 (34) orthogonal experiments were also applied to optimize the DRL preparation conditions. Firstly, PVA (1.5–2.5 g) was dissolved in 20 mL deionized water at 95 °C and stirred for 0.5 h. After that, the temperature was turned down to 65 °C, and β-CDP (1.00–1.50 g) and anti-inflammatory drug DS (0.20–0.30 g) were added into the solution and stirred for another 0.5 h to obtain the hydrogel precursor solution. After cooling to room temperature, the solution was poured into the mold and preserved at −20 °C for 3 h. Then, the sample was taken out and balanced at room temperature for 1 h. The DRL was obtained by 1–3 freeze–thaw cycles. The amounts of each polymer monomer and freeze and thaw time are listed in Table 6.

Double layer (DL): In the mold, 2 mL of deionized water was uniformly added to the surface of MSL. Afterward, the sample was sealed and stored at room temperature for 0.5 h. Finally, the solution of the DRL was poured onto the MSL, and the rest of the process was the same as the preparation of DRL. The sample preparation process is schematically revealed in Scheme 1b.

**Table 6:** Level factor of the orthogonal experiment of DRL.

Level	A (PVA/g)	B (β-CDP/g)	C (DS/g)	D (Freeze–Thaw Times)
1	1.50	1.00	0.20	1
2	2.00	1.25	0.25	2
3	2.50	1.50	0.30	3

### 3.5. Determination the Solubility, Polymerization Degree, and Activity of β-CDP

The solubility of β-CDP in water was measured at 25 °C. Plenty of β-CDP was added to 5 mL of deionized water to reach saturation. Then, the solution was stirred at 25 °C for 2 h, and the undissolved solid material was filtered out from the solution. The β-CDP on the filter paper was further dried in a vacuum drying oven until the mass of the substance was constant. The solubility of the β-CDP was determined by the differences between the amount of original β-CDP added and the amount of the β-CDP left on the filter paper. All assays were performed three times.

The degree of polymerization and the average relative molecular weight of the β-CDP were determined at 40 °C by gel penetration chromatography (GPC) (1100 HPLC, Agilent, Palo Alto, CA, USA).

The activity of β-CDP was detected by PP probe technology [39]. The testing principle is that the PP solution becomes colorless due to the inclusion of associations between the β-CD and PP molecules (the stoichiometry ratio is 1:1) at pH 9.18. The detailed procedures are given in the Supporting Information.

The contents of active monomers were calculated by the regression linear relationship of the absorbance difference between the β-CD blank (pure PP solution) and their corresponding PP/β-CD complex solutions (ΔA) against $C_{\beta -\mathrm{CD}}$. The content of β-CD ($C_{\beta -\mathrm{CD}}$) was calculated with Equation (2):

(2) $$\Delta A=1.44875C_{\beta -\mathrm{CD}}+0.01138$$

where $C_{\beta -\mathrm{CD}}$ denotes the concentration of β-CD (mM).

Then, the sample of PP/β-CDP was prepared by the above method (0.20 mL β-CD solutions (1 × 10^−3^ M) and 1.00 mL of PP solution (1 × 10^−3^ M)). The proportion of active monomers to total monomers of β-CDP was obtained by Equation (2). Here, ΔA was calculated as the absorbance differences between the solutions of β-CD blank sample and PP/β-CDP. Finally, the activity of β-CDP was calculated by Equation (3):

(3) $$\mathrm{activity}\;\mathrm{of}\;\beta -\mathrm{CDP}=\frac{C_{\beta -\mathrm{CDP}}}{0.004\times 5}\times 100\%$$

where $C_{\beta -\mathrm{CDP}}$ denotes the concentration of β-CDP (mM).

Finally, the drug loading capacity per unit volume of water was calculated by Equation (4):

(4) $$\mathrm{drug}\;\mathrm{loading}\;\mathrm{capacity}=\frac{m}{M}\times \mathrm{degree}\;\mathrm{of}\;\mathrm{polymerization}\times \mathrm{activity}\;\mathrm{of}\;\beta -\mathrm{CDP}$$

where m denotes the weight of β-CD or β-CDP in saturated solution per unit volume (g), and M denotes the molar mass of β-CD or β-CDP (g mol^−1^).

### 3.6. Static Tensile Test of DL Hydrogel

The maximum tensile strength and the elongation degree to break were tested using an electronic universal testing machine (SHIMADZU, model AGS-X, 100 N,Tokyo Japan) with a tensile loading rate of 0.2 mm s^−1^. All specimens were cut into a specific dumbbell shape (50 mm long, 4 mm width at the middle part, and 3 mm thickness of measuring segment) and performed in a dry state.

### 3.7. Drug Release Analysis of DL Hydrogel

The in vitro drug release kinetics of DS loaded hydrogels were studied by the solution immersion method. In brief, a small part (5 cm × 5 cm × 3 cm) of each sample was immersed in a 50 mL PBS buffer (pH = 7.4). At set intervals, 1 mL of the solution was withdrawn and replenished with the same amount of fresh buffer solution to maintain a constant volume release medium. Subsequently, the solutions were diluted and measured by a UV–Vis spectrophotometer at 264 nm.

In order to analyze the mode of drug release, the release data were analyzed by the typical mathematical models: Ritger–Peppas model (Equation (5)), Peppas–Sahlin model (diffusion–relaxation model) (Equation (6)), and diffusion–relaxation–erosion model (Equation (7)) [40,41,42]:

(5) $$\frac{M_{t}}{M_{\infty }}=kt^{n}$$

(6) $$\frac{M_{t}}{M_{\infty }}=k_{1}t^{m}+k_{2}t^{2m}$$

(7) $$\frac{M_{t}}{M_{\infty }}=k_{1}t^{m}+k_{2}t^{2m}+k_{3}t^{4m}+k_{4}t^{6m}$$

where $M_{t}$ is the weight of drug release at time *t*, $M_{\infty }$ is the weight of drug release at infinite time, $k,\;k_{1},\;k_{2},\;k_{3},\;k_{4}$ are the rate constant and correlation coefficients, and n, m represent the diffusion index, which is related to the drug release mechanism. When *n* < 0.45, the drug release mechanism is followed by Fick diffusion; when *n* > 0.45, it does not comply with Fick diffusion [27].

### 3.8. Rheological Measurements

Dynamic rheological tests were performed using a Modular Compact Rheometer (Anton Paar MCR 302, Anton Paar, Ostfildern, Germany). Each hydrogel sample was cut into a circular disk with a diameter of 25 mm. Dynamic strain scanning experiments were carried out on the hydrogels to determine the linear viscoelastic region (0.01% to 10%). Then, frequency sweep tests were performed in the frequency range 0.1–100 rad s^−1^ at a strain of 1% for all samples.

### 3.9. Determination of Water Retention

The water retention properties of the MSL, DL, and DRL hydrogel were tested at room temperature and 65% RH of indoor humidity. The test times were 0.5, 1, 2, 4, 6, 8, 10, 12, 24, 36, and 48 h. The water retention rate *(*$W_{r}$) of hydrogels was determined according to the following Equation (8):

(8) $$W_{r}=\frac{W_{t}}{W_{0}}\times 100\%$$

where $W_{0}$ and $W_{t}$ are the initial weight of the hydrogel and the weight of the hydrogel at time t, respectively.

## 4. Conclusions

In this study, we developed a DL hydrogel dressing composed of MSL and DRL based on the orthogonal experiments. For the MSL, immersing calcium chloride/boric acid could effectively improve the mechanical strength of the hydrogel due to the formation of boron ester bonds and ionic bonds, and increasing polymer chain density could further endow the hydrogel dressing with excellent water retention. For the DRL, adding β-CDP reduced the hydrogel’s drug release rate and prolonged the drug release time. Experimental results showed that our DL hydrogel dressing possessed favorable mechanical properties, water retention capacity, skin adhesion property, and drug sustained release property. In addition, the distinctive drug release curve of the DL hydrogel agreed with the diffusion–relaxation–corrosion model, indicating that the DL hydrogel is utterly different from the monolayer hydrogel in the drug release mechanism. Thus, we believe that the DL hydrogel could retain more moisture and provide sufficient mechanical strength while maintaining the slow release of drugs, indicating its potential as a hydrogel dressing.

## Figures and Tables

**Table 1:** Estimated parameters k and n obtained by experimental data and equation.

Samples	k	n	$R^{2}$
Without β-CDP	0.88	0.036	0.9972
With β-CDP	0.64	0.14	0.9717

**Table 2:** Related estimated parameters obtained by above experimental data and equation.

Model	*k*	n	$k_{1}$	$k_{2}$	$k_{3}$	$k_{4}$	$R^{2}$
Ritger–Peppa model	0.2868	0.2072	-	-	-	-	0.5963
Diffusion–relaxation model	-	-	0.3166	0.04741	-	-	0.7944
Diffusion–relaxation–corrosion model	-	-	0.03668	0.1558	0.01459	0.00036	0.9902

**Table 3:** Diffusion exponent and solute release mechanism.

Diffusion Exponent (m)			Mechanism
Film	Cylinder	Sphere	
0.50	0.45	0.43	Fickian diffusion
0.50 < m < 1.00	0.45 < m < 0.89	0.43 < m < 0.85	Anomalous transport
1.00	0.89	0.85	Case Ⅱ transport

**Table 4:** Summary and comparison of other hydrogels’ properties.

Matrix	Structure and Morphology	G′	Water Retention	Drug Releasing Kinetic
AA, HEMA, AAm	Single-layer film	10^4^ Pa	-	Ritger-Peppas model [21]
CHI, ACHI, PA	Double-layer film	10^2^ Pa	-	-[33]
Ca-Alg	Single-layer sphere	-	-	Diffusion-relaxation model [34]
CS, TPP, HA	Multiple-layer sphere	-	-	Ritger-Peppas model [35]
Gel, γ-PGA	Single-layer film	-	48 h, 37%	-[36]
CS, cuminaldehyde	Single-layer film	10^3^ Pa	-	-[37]
This work	Double-layer film	2.5 × 10^4^ Pa	48 h, 67%	Diffusion-relaxation-erosion model

**Table 5:** Level factor of the orthogonal experiment of MSL.

Level	A (PVA/g)	B (CMC/g)	C (Soaking Time/h)
1	2.50	0.10	0.50
2	3.00	0.20	1.00
3	3.50	0.30	1.50

## Data Availability

Data available on request from the authors.

## Supplementary Materials

The following supporting information can be downloaded at: https://www.mdpi.com/article/10.3390/molecules28020499/s1, Table S1: Arrangements and analysis of the 3-variable 3-level orthogonal experiment of MSL hydrogels.; Table S2: Arrangements and analysis of the 4-variable 3-level orthogonal experiment of DRL hydrogels.; Figure S1. Water loss display of hydrogel in different time periods.
