# Supplementary material for: A Double-Layer Hydrogel Dressing with High Mechanical Strength and Water Resistance Used for Drug Delivery
Source: Molecules. 2023 Jan 4;28(2):499. doi: 10.3390/molecules28020499 (PMC9864262; doi:10.3390/molecules28020499)
Supplement: Supplementary file 1 [file molecules-28-00499-s001.zip › molecules-2099791-supplementary.pdf]

Supporting Information

Experimental section

Determination of the β-CDP activity

Briefly, the standard curve for the concentration of β-CD and the absorbance of PP solution was quantified with the photometric titration method. The β-CD solutions ($1x10^{-3}$ M) of 0.00, 0.20, 0.60, 1.00, 1.40, 1.80 and 2.00 mL were added separately to 50 mL volumetric flasks. After that, each flask was added with 1.00 mL of PP solution ($1x10^{-3}$ M). Then, all flasks were diluted with buffer solution (pH = 9.18) to 50 mL. After 24 h, they were examined with a UV–Vis spectrophotometer, and the absorbance of the calibration solutions of β-CD were recorded at a wavelength of 553 nm in room temperature. The contents of active monomers were calculated by the regression linear relationship of the absorbance difference between the β-CD blank (free PP solution) and their corresponding PP/β-CD complex solutions (ΔA) against C_{CD}. The content of β-CD (C_{CD}) was calculated with Equation (1):

$$\Delta A = 1.44875 C_{CD} + 0.01138 \qquad (1)$$

Where C_{CD} denotes the concentration of β-CD (mM).

Then, the sample of PP/β-CDP was prepared by the above method (0.20 mL β-CD solutions ($1x10^{-3}$ M) and 1.00 mL of PP solution ($1x10^{-3}$ M)). And the proportion of active monomers to total monomers of β-CDP was obtained by Equation (1). Here, ΔA was calculated as the absorbance differences between the solutions of β-CD blank sample and PP/β-CDP. Finally, the activity of β-CDP was calculated by Equation (2):

$$\text{activity of } \beta - \text{CDP} = \frac{C_{\beta-CDP}}{0.004 \times 5} \times 100\ \% \qquad (2)$$

Table S1. Arrangements and analysis of the 3-variable 3-level orthogonal experiment of MSL hydrogels.

Test number	Component (ABC)			stress strength(kPa)
	PVA(A)	CMC(B)	Soaking time(C)	
1	2.5 g	0.1 g	0.5 h	425.500
2	2.5 g	0.2 g	1.0 h	1100.839
3	2.5 g	0.3 g	1.5 h	864.668
4	3.0 g	0.1 g	1.0 h	1084.851
5	3.0 g	0.2 g	1.5 h	1690.107
6	3.0 g	0.3 g	0.5 h	1412.653
7	3.5 g	0.1 g	1.5 h	1622.714
8	3.5 g	0.2 g	0.5 h	1393.033
9	3.5 g	0.3 g	1.0 h	1460.458
K1	2391.007	3133.065	3231.186	
K2	4187.611	4183.979	3646.148	
K3	4476.205	3737.779	4177.489	
k1	797.002	1044.355	1077.062	
k2	1395.870	1394.660	1215.383	
k3	1492.068	1245.926	1392.496	
Range R	695.066	350.305	315.434	
Optimal level	A_3(3.5 g)	B_2(0.2 g)	C_3(1.5 h)	

Table S2. Arrangements and analysis of the 4-variable 3-level orthogonal experiment of DRL hydrogels.

Test number	Component（CABD）				Sustained release time (The time of 85 % release)	Releasing amount	Score
	PVA(A)	β-CDP(B)	DS(C)	Freez-thaw times (D)			
1	1.5 g	0.1 g	0.02 g	1	6 h	0.948	0.641
2	1.5 g	0.2 g	0.04 g	2	4 h	0.922	0.544
3	1.5 g	0.3 g	0.06 g	3	6 h	0.892	0.571
4	2.0 g	0.1 g	0.04 g	3	2 h	0.968	0.526
5	2.0 g	0.2 g	0.06 g	1	2 h	0.929	0.506
6	2.0 g	0.3 g	0.02 g	2	4 h	0.908	0.537
7	2.5 g	0.1 g	0.06 g	2	2 h	0.963	0.523
8	2.5 g	0.2 g	0.02 g	3	6 h	0.929	0.589
9	2.5 g	0.3 g	0.04 g	1	2 h	0.920	0.501
K1	1.756	1.689	1.767	1.648			
K2	1.569	1.640	1.571	1.605			
K3	1.614	1.610	1.600	1.686			
k1	0.585	0.563	0.589	0.549			
k2	0.523	0.547	0.524	0.535			
k3	0.538	0.537	0.533	0.562			
Range R	0.062	0.026	0.065	0.027			
Optimal level	A_1(1.5 g)	B_1(0.1 g)	C_1(0.02 g)	D_3(3 times)			

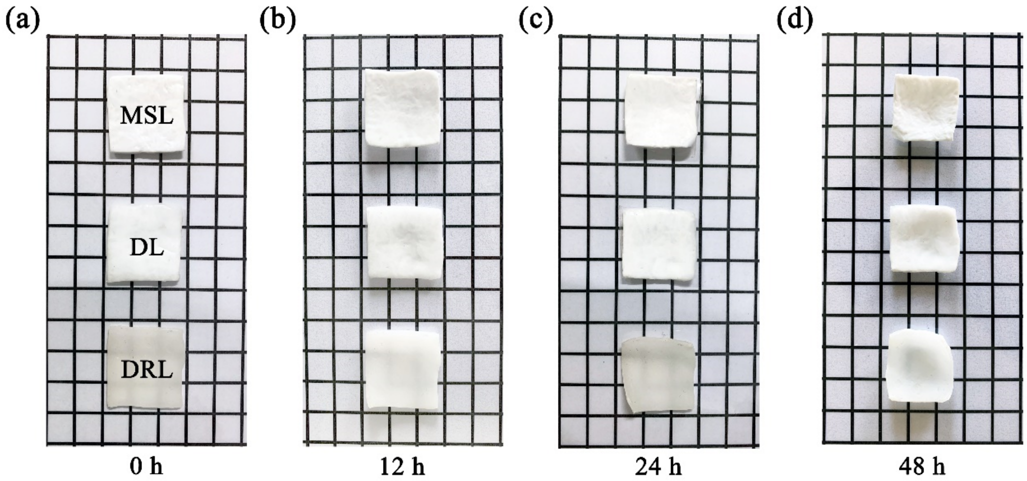

Figure S1. Water loss display of hydrogel in different time periods.
